# Liquid Crystalline Copolymers Containing Sulfonic and Light-Responsive Groups: From Molecular Design to Conductivity

**DOI:** 10.3390/molecules25112579

**Published:** 2020-06-02

**Authors:** Sakinah Mohd Alauddin, Nurul Fadhilah Kamalul Aripin, Thamil Selvi Velayutham, Alfonso Martinez-Felipe

**Affiliations:** 1 Faculty of Chemical Engineering, Universiti Teknologi MARA, Shah Alam 40450, Selangor Darul Ehsan, Malaysia; sakinah3676@uitm.edu.my (S.M.A.); fadhilah9413@uitm.edu.my (N.F.K.A.); 2 Fundamental and Frontier Sciences in Nanostructure Self-Assembly Center, Department of Chemistry, Faculty of Science, University of Malaya, Kuala Lumpur 50603, Malaysia; t_selvi@um.edu.my; 3 Low Dimensional Material Research Center, Department of Physics, Faculty of Science, University of Malaya, Kuala Lumpur 50603, Malaysia; 4 Chemical and Materials Engineering Research Group, School of Engineering, University of Aberdeen, King’s College, Old Aberdeen AB24 3UE, UK

**Keywords:** liquid crystalline polymers, light-responsive materials, ionic conductivity, polymer electrolytes, azobenzenes

## Abstract

In the search for novel smart multifunctional liquid crystalline materials, we report the synthesis, thermal and structural characterisation, and the conductivity, of a set of new block and statistical copolymers, containing light-responsive mesogenic groups (MeOAzB), polar sulfonic acids (AMPS), and methyl(methacrylate) groups (MMA). By using a cascade of reversible addition-fragmentation chain polymerisations, RAFT, we have tailored different side-chain polymeric structures by controlling monomer composition (MeOAzB/AMPS/MMA) and configuration. We have yielded simultaneous liquid crystalline behaviour and appreciable conductivity in polymers with low concentrations of polar acid groups, by the formation of smectic phases in narrow aggregates. The light-responsiveness of the polymers, via reversible *trans*-to-*cis* photoisomerization of azobenzene groups, and the local activation of conductivity at relatively low temperatures, opens the possibility to prepare polymer electrolytes for energy conversion and storage, whose conductivity could be controlled and optimised by external stimuli, including light irradiation.

## 1. Introduction

Liquid crystals, LCs, are fascinating materials that exhibit intermediate states of molecular order between isotropic liquids and crystalline solids, and continue to attract the interest of researchers and industrialists. [1,2] Through the formation of mesophases, liquid crystals combine molecular mobility and long-range order, and their anisotropic properties can be tuned by the application of external fields. Liquid crystals were discovered by Reinitzer at the end of the XIX century [3], and a great variety of liquid crystalline materials exist nowadays, nurturing the development of basic sciences and applied technologies [4]. Nematic liquid crystals are considered to be the simplest LC mesophases and consist of mesogenic rod-like molecules with flexible terminations capable of forming strong anisotropic interactions and relatively low melting points, and have been the main focus of technological interest since the 1970s, used in flat displays, fibres or optical films [5]. Almost simultaneously, discotic materials, with two-dimensional anisotropy, have been extensively studied since the seminal work by Chandrasekhar et al. in 1977, and are primarily used as optical compensating films [6]. 

The advance in covalent synthesis and supramolecular assembly techniques (such as, hydrogen-bonding [7] or ionic interactions) have promoted the proliferation of a variety of molecular structures to show liquid crystallinity, very often inspired in complex motives already existing in nature [8]. These include, not only calamitic and discotic, but also bent-core, dimeric, cholesteric, oligomeric, and polymeric materials, as well as crosslinked networks. The interplay between molecular geometry, intermolecular forces and packing efficiency, allows to anticipate structure-properties relationships, and to investigate non-conventional mesophases and new applications. Thermotropic [9] and lyotropic [10] liquid crystals are forefront components used in nanotechnology disciplines [11], as they are responsive materials to light [12], mechanical [13] or electrical [14] fields; serve as components in separation membranes [13,15], sensors [16], actuators [17,18], templates and self-assemblies in bio-applications [19] including DNA gene therapy [20,21]; or serve as field effect transistors, light-emitting devices, photovoltaic devices, and electronic noses [22].

In particular, polymeric electrolytes with liquid crystalline nanostructures are very attractive candidates to optimise transport of ionic or electronic charges [23]. The formation of soft liquid-crystalline phases, but stable under a certain range of temperatures, can promote ionic transport by a combination of short-range mobility and long-range anisotropy [15]. In addition, their responsive character results in the exciting possibility to control transport by external stimuli, such as the application of electric fields, mechanical shear or light irradiation. Transport through well-organised and aligned channels can lead to efficient anhydrous proton conductivity via ion hopping, thus reducing the dependence of conductivity on the presence of water in some energy conversion devices [24]. Removing water and other solvents from polymer electrolytes in fuel cells and batteries will allow them to operate at higher temperatures without evaporation concerns [25,26,27], to use cheaper catalysts that are less sensitive to poisoning (e.g., to CO), and to reduce undesired fuel crossover from anode to cathode. [28,29] In the long term, the development of dry electrolytes will help commercialise fuel cells (and battery) technologies and will ultimately contribute to decarbonise power sources and supply.

Under these principles, several liquid crystalline electrolytes with 1D-columnar, 2D-planar and 3D-bicontinuous morphologies have been designed and evaluated as components of energy conversion and storage devices [30]. That is the case of poly(imides) with planar alignments [31] and electrolytes with columnar nanostructures [32], which present considerable proton conductivity necessary for fuel cells, or discotic materials that inhibit the crystallisation of poly(ethylene oxide), thus increasing lithium mobility in batteries [33]. Current liquid crystalline polymeric electrolytes, however, still have low values of conductivity compared to other non-mesogenic polymers containing, for example, poly(sulfonic) and poly(aromatic) backbones [34,35]. Whilst the introduction of polar groups is necessary to achieve ion transport, their presence in high concentrations can inhibit the formation of liquid crystalline phases due to the strong interactions with neighboring mesogenic groups. It is then necessary to develop new materials where functional groups in the nanoscale proximity retain their respective functionalities [36].

In the search to achieve anhydrous ionic conductivity, during the past few years we have prepared and characterised new liquid crystalline copolymers containing sulfonic groups [37,38,39,40]. In a recent contribution, we synthesised statistical side-chain terpolymers capable to form smectic phases with promising conductivity values in anhydrous conditions [37]. The use of conventional radical polymerisation, however, resulted in polymers with limited control over their chain compositions. In addition, considerably high amounts of sulfonic groups were needed to achieve conductivity (>30%, mol%), which distorted the liquid crystal behaviour and rose the polydispersity values to Mw/Mn > 3. We explained this effect, in part, by the different reactivity ratios of sulfonic and azobenzene-based monomers [41,42,43].

In the present work, we aim to prepare new liquid crystalline block copolymers with ionic conductivity, by using reversible addition-fragmentation chain transfer polymerisation, RAFT [44], as a way to control the distribution of the different monomers in the polymer chains. More specifically, we have combined light-responsive azobenzenes, polar sulfonic groups and methyl(methacrylate) monomers. Block copolymers offer a very versatile strategy to tailor the properties of the new electrolytes, by locating specific functionalities in different segments [45,46,47]. We envisage that the application of controlled radical polymerization techniques will facilitate the simultaneous introduction of polar and light-responsive units in new electrolytes, which is normally challenging, due to their very different chemical natures [48,49,50]. We envisage that the new multifunctional materials prepared in this work will open new possibilities to develop controllable polymeric liquid crystalline electrolytes with anhydrous conductivity, but also sensors and light-responsive materials with high spatial and temporal resolution, via the introduction of photochromism [51].

## 2. Results and Discussion

### 2.1. Synthesis and Thermal Stability of the Polymers and Copolymers

**Figure 1:**
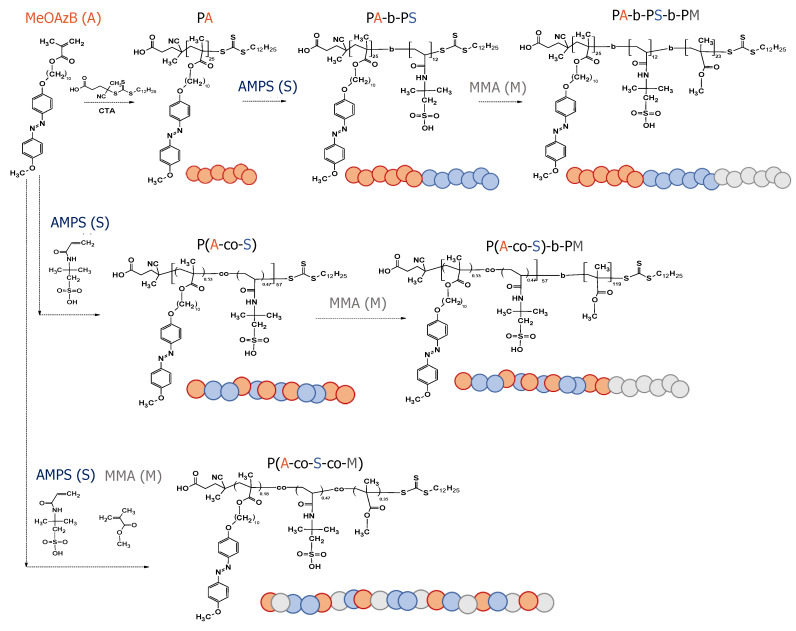
Chemical structure of the MeOAzB (**A**), AMPS (**S**) and MMA (**M**), monomeric units; synthetic routes of the polymers and copolymers prepared, and schematic representation of their monomers distributions.

The polymeric materials prepared in this work and their simplified synthetic routes are summarised in Figure 1. We have used three different monomeric units: (i) 10-(4-methoxyazobenzene -4′-oxy)decyl methacrylate (MeOAzB) as the mesogenic group (**A**); (ii) acrylamido-2-methyl-1-propanesulfonic acid (AMPS) as a polar group to facilitate ionic conduction (**S**); (iii) methyl(methacrylate) (MMA) as a non-mesogenic/non-ionic structure modifying unit (**M**).

In Table 1 we label the polymers, together with their composition, number average molecular weight and weight average molecular weight (Mn and Mw, respectively), polydispersities (Mw/Mn), and yields. Fractional compositions of statistical polymeric blocks were estimated by ^1^H-NMR, by calculating the relative integrals of the 7–8 ppm signals, assigned to the phenyl azobenzene protons (8H) of MeOAzB; the ~2.7 ppm singlet, assigned to the methylene groups (2H) adjacent to the sulfonic acid groups in AMPS; and the singlet at ~3.6 ppm, associated with the methyl groups (3H) of MMA; see Figure S2 as an example. The lengths of the blocks were estimated by GPC/SEC, and, when possible, were confirmed by integrating the ^1^H-NMR signals corresponding to the monomeric units and the CTA. The ^1^H-NMR plots of polymers and their intermediates are shown in Figure S3.

Three macro-CTAs corresponding to the homopolymers PMeOAzB_25_ (PA), PAMPS_192_ (PS) and PMMA_136_ (PM), were prepared as building blocks. A second block was then attached to PMeOAzB_25_ by polymerising AMPS monomeric units, leading to PMeOAzB_25_-*b*-PAMPS_12_ (PA-b-PS). The diblock copolymer (PA-b-PS) was further polymerised to attach MMA units, leading to the triblock copolymer PMeOAzB_25_-*b*-PAMPS_12_-*b*-PMMA_23_ (PA-b-PS-b-PM).

With the aim to investigate the effect of block and statistical configurations, we have also prepared (by RAFT polymerisation) a fourth macro-CTA corresponding to the statistical copolymer P(MeOAzB_0.53_-*co*-AMPS_0.47_)_57_ (P(A-co-S)), to which we have then grown a second MMA block to yield P(MeOAzB_0.53_-*co*-AMPS_0.47_)_57_-*b*-PMMA_119_ (P(A-co-S)-b-PM). For the sake of comparison, we have synthesised one statistical terpolymer containing the three monomeric units, P(MeOAzB_0.18_-*co*-AMPS_0.47_-*co*-MMA_0.35_), also obtained by RAFT polymerisation, P(A-co-S-co-M). We note that the structures of P(A-co-S) and P(A-co-S-co-M) can be compared to our previous materials studied in [40] and [37], respectively. Diblock copolymers PMeOAzB_25_-*b*-PMMA_22_ (PA-b-PM) and PMMA_136_-*b*-PAMPS_471_ (PM-b-PS) have been prepared as reference materials, by polymerising a second monomer to the corresponding macro-CTAs, PA and PM.

Whilst PA, PS and PM show low polydispersity ratios (Mw/Mn between 1 and 2), the dispersity of the chains’ distribution increases upon copolymerisation, as it was expected. We note, however, that our polymers have reasonable Mw/Mn values (Mw/Mn < 2.1) and high molecular weights, in the 10–40k g mol^−1^ range. The addition of AMPS increases both the molecular weight and polydispersity, but it is noteworthy that our Mw/Mn values are smaller than other copolymers with comparable (moderate and high) sulfonic groups concentrations [37,40,52,53,54]. This fact is particularly challenging, considering the different reactivity rates of the three monomers involved [41,42,43]. Polymers with statistical blocks containing the three monomeric units, P(A-co-S)-b-PM and P(A-co-S-co-M), possess the highest polydispersities within the series (Mw/Mn ≥ 1.83), but their last synthetic steps also reach the highest yields (>70%).

**Figure 2:**
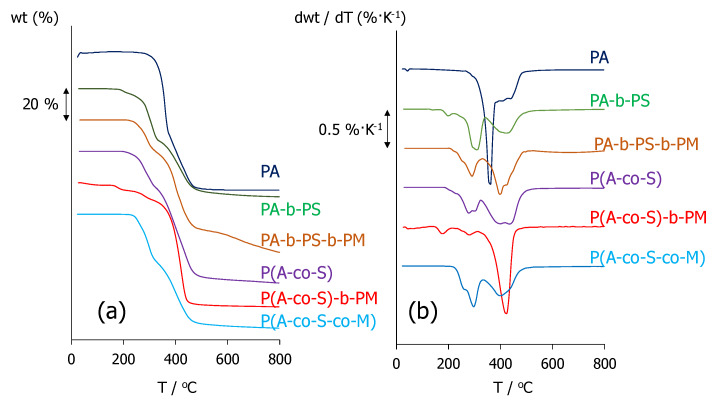
(**a**) Thermogravimetric (TG) and (**b**) derivative thermogravimetric (DTG) curves of polymers containing MeOAzB and AMPS groups. The mesogenic homopolymer (PA) has been included for the sake of reference. Curves have been shifted arbitrarily along the Y-axes.

The thermal stability of the polymers was assessed by thermogravimetric analysis, TGA, and Figure 2a,b display their thermogravimetric (TG) and derivative thermogravimetric (DTG) curves, respectively. Thermal degradation of the copolymers initiates with a process about 300 °C, which can be attributed to decomposition of lateral units, followed by a weight loss around 400 °C, related to loss of the main chain [55,56]. The introduction of AMPS and MMA units seems to increase the liability of the materials, but all polymers exhibit considerably high thermal stabilities under inert conditions, and their temperatures for 2% weight loss, T_2%_, are above 170 °C. These results are in excellent agreement with the degradation profiles and mechanisms reported in materials with comparable compositions [37], and confirm that the present polymers could be used under a broad range of temperatures.

### 2.2. Phase Behaviour

**Figure 3:**
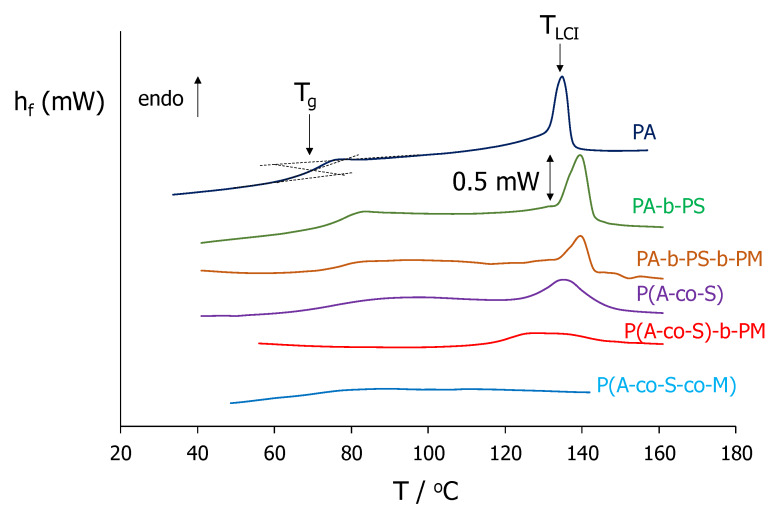
Differential scanning calorimetry (DSC) thermograms obtained in the second heating scan of polymers containing MeOAzB and AMPS groups. The mesogenic homopolymer (PA) has been included for the sake of reference. Curves have been shifted arbitrarily along the Y-axis (h_f_, heat flow).

The phase behaviour of the copolymers under study was determined by polarised optical microscopy (POM) and differential scanning calorimetry (DSC), and the thermal parameters are summarised in Table 2. The formation of liquid crystalline phases was assessed by the appearance of fluid birefringent textures when viewed through the polarised optical microscope, on cooling from the isotropic phases. Unfortunately, the textures were not well-defined, and we could not assign the mesophases unambiguously, which is normally occurring in highly viscous liquid crystalline polymers, see Figure S4. Our POM observations were in excellent consistency with the DSC thermograms, and in Figure 3 we show the curves corresponding to the polymers containing MeOAzB and AMPS units.

PA-b-PS, PA-b-PS-b-PM and P(A-co-S) form liquid crystalline phases on cooling from their isotropic phases, before vitrifying at sufficiently low temperatures. P(A-co-S)-b-PM and P(A-co-S-co-M), on the other hand, are amorphous materials, and form glassy phases below their respective glass transitions, T_g_. It is possible to observe some additional weak transitions in the P(A-co-S)-b-PM thermogram in Figure 3, but the high concentration of MMA units in the second block may preclude the mesogenic behaviour of this copolymer.

According to the compositions in Table 1, a minimum amount of ~40% MeOAzB (mol%, rod-like azobenzene units) is needed to form mesophases, which is in good agreement with thermal studies previously reported for MeOAzB-based materials [37,40]. For the MeOAzB homopolymer, PA, we have obtained values of T_LCI_ = 130.1 °C (liquid crystal to isotropic transition temperature) and T_g_ = 67.7 °C (glass transition) [37,39,40]. Our current results suggest that the liquid crystal range is maintained when the MeOAzB units are included as segments of PA-b-PS and PA-b-PS-b-PM, with a slight increase in liquid crystal stability (T_LCI_ ~ 139 °C), accompanied with a rigidising effect on the polymer chain (T_g_ ~ 78 °C). These results are relevant and prove that we have introduced mesogenic functionality in our materials.

### 2.3. Phase Structure

**Figure 4:**
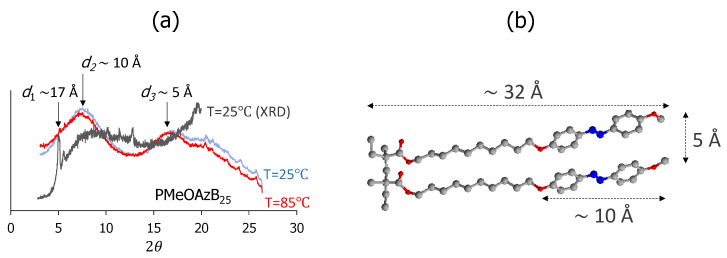
(**a**) SAXS scatterings (in the liquid crystal phase, red curve at T = 85 °C; and in the glass phase, blue curve, T = 25 °C) and XRD diffractogram (grey curve, T = 25 °C) of the mesogenic homopolymer PA, highlighting relevant reflections and their corresponding distances; (**b**) molecular model of two contiguous MeOAzB side chains.

Figure 4 shows the X-ray diffraction (XRD) and small angle X-ray scattering (SAXS) curves of PA, which are consistent with the formation of smectic phases in this homopolymer. Its XRD curve obtained at room temperature exhibits a sharp small-angle scattering peak, 2θ_1_, which indicates long-range lamellar order in the glass phase. This reflection corresponds to the smectic periodicity of d_1_ ~ 17 Å, which is roughly half the length of the MeOAzB side chains in all-*trans* conformations, and a secondary peak is also visible at d’_1_ ~ 15.81 Å. This latter peak was interpreted in the past by the formation of fully interleaved smectic A phases (SmA_1_), with quasi-symmetrical distributions of the electronic density about the mid-point of the smectic layers. [57,58] The prominence of the former peak, d_1_, indicates the formation of larger domains, and may reflect a more effective stacking between azobenzene groups across the smectic layer, as will be further discussed later.

In the SAXS traces, the reflection associated to the smectic periodicity at 2θ_1_ appears with weaker intensity than in the XRD diffractions, and overlapped with contributions at wider angles. A broad reflection is also visible, 2θ_2_, which fits to the length of the azobenzene group, d_2_ ~ 10 Å, as well as a reflection at wider angles, 2θ_3_, which we attribute to the periodicity along the polymer backbone, d_2_ ~ 5 Å. We note that the temperature has limited effect on the signals (compare blue and red curves in Figure 4) confirming that the liquid crystalline order is maintained (vitrifies) at temperatures below the glass transition.

**Figure 5:**
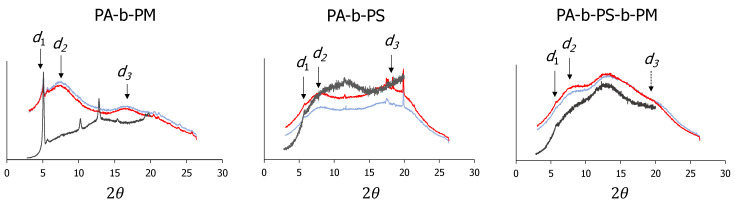
SAXS scatterings (obtained at 85 °C, red; at room temperature, 25 °C, blue) and XRD diffractograms (obtained at room temperature, 25 °C, grey) of the block copolymers forming liquid crystalline phases, highlighting relevant reflections related to the spacings found in Figure 4, d_i_. Y-axes correspond to the diffractogram intensity, a.u.; and curves have been shifted arbitrarily along this axis. We have indicated in the PA-b-PS-b-PM curve the underlying d_3_ spacing associated to the distance between azobenzene units in the copolymer chains.

The diffractograms of the copolymers also display similar reflections as those of PA, but with some shifts in their shapes and maxima, due to the introduction of new units, and we show some examples in Figure 5. The XRD diffraction patterns obtained at room temperature for all the polymers prepared in this study are depicted in Figure S5, and the reflections measured from the XRD or SAXS signals at room temperature are summarised in Table 3.

PA-b-PM illustrates the confinement of the MeOAzB units in a block copolymer and has very similar molecular reflection values as PA. The inclusion of AMPS and MMA groups, on the other hand, promotes the appearance on new reflections associated to different monomeric units, and it is noteworthy that in PA-b-PS and PA-b-PS-b-PM, there are signals that can be associated to the two and three blocks, respectively. The XRD curves obtained for statistical polymers, on the other hand, show stronger deviations from the pristine homopolymers (PA, PS and PM), see also Figure S5, and we explain this effect by interactions between unlike units in the same polymer segments [59].

**Figure 6:**
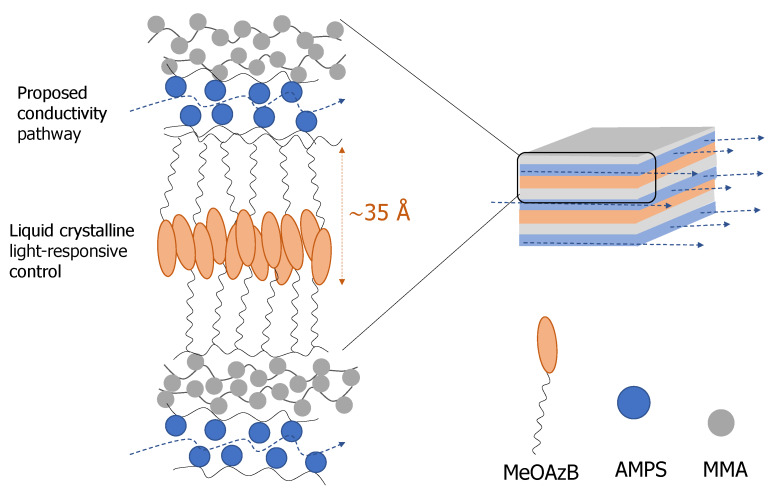
Schematic representation of the micro-segregated structure of the triblock copolymer, PA-b-PS-b-PM, including liquid crystalline regions and potential ionic conductivity pathways (dotted lines).

The liquid crystalline layers in the block copolymers PA-b-PS and PA-b-PS-b-PM seem to correspond to the smallest distance observed in the PA homopolymer diffractogram (d’_1_ ~ 15.81 Å), and TEM micrographs suggest the formation of narrow liquid crystalline nanodomains, d_0_ ~ 50/60 Å, see Figure S6. Considering the XRD/SAXS results in Table 3, this could correspond to smectic layers with very limited interdigitation of the mesogenic units, see Figure 6. With such narrow domains we would expect to have strong interfacial interactions between blocks, and this could also explain the low ∆H_LCI_ and ∆S_LCI_/R values measured for the copolymers in Table 2, compared, for example, with PA. Another interesting observation is the formation of continuous lamella domains extending through the microstructure of the block copolymers, and we will return to this observation later.

### 2.4. Light-Responsive Behaviour

**Figure 7:**
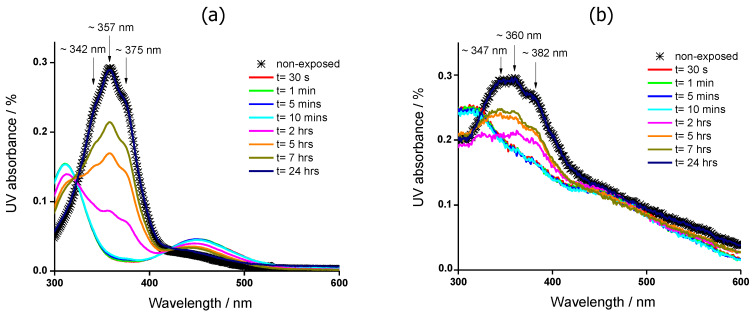
UV-visible spectra of PA-b-PS-b-PM, measured at room temperature, obtained for a: (**a**) 3.73 10^−5^ M THF solution; (**b**) film cast on a quartz substrate. Crosses correspond to the original spectrum prior to UV exposure (non-exposed), and dotted arrows indicate the *cis-*to*-trans* thermal relaxation with time, *t*, after exposure, while keeping the samples in the dark. Solid arrows indicate contributions of non-aggregated (~357/360 nm) and aggregated (~342/347 and ~375/382 nm) azobenzenes.

In Figure 7a,b we show the UV-visible spectra of PA-b-PS-b-PM, obtained in a ~3.73 × 10^−5^ M THF solution and in a quartz film, respectively, as illustrative of other materials in the series containing MeOAzB chains. Two absorption regions are visible and are associated to transitions of the azobenzene groups: an intense UV band centred at ~357 nm is related to the π-π* transition, and a weak and broad absorption band at ~450 nm is related to the symmetric forbidden *n*-π* transition [60]. These correspond to energetic transitions of the *trans* isomer of non-aggregated azobenzenes. Additionally, we also distinguish some shoulders in the π-π* transition band which denote the existence of H- and J-aggregates that are expected at around 342 nm and 375 nm, respectively [61]. Comparable results are obtained for the rest of the copolymers containing MeOAzB units and confirm that their light response takes place via photo-isomerisation of azobenzene groups.

Upon irradiation with UV light at 365 nm, the azobenzene groups undergo *trans*-to-*cis* isomerisation, and, as a result, the absorption band π → π* shifts to shorter wavelengths by a hypsochromic effect (~320 nm), and simultaneously decreases in intensity. In the *cis* form, the azobenzene groups are allowed to undergo the electronic transition n → π*, which promotes an increase of the ~450 nm band, with respect to the *trans* isomer [62]. When the samples are kept in the dark, the intensity of the π-π* band increases with time, whereas that of the n-π* band decreases. After 24 h, the original UV absorption spectra, see Figure 7, are essentially recovered in all the samples, due to the thermally activated *cis*-to-*trans* relaxation.

There are some differences in the photo-response of the polymers when measured as films and in THF solutions. The spectra obtained from the films show stronger shoulders with respect to the ~360 nm maximum, see Figure 7b for PA-b-PS-b-PM, denoting the formation of more H- and J-aggregates than in the solution, see Figure 7a. We also note that the π-π* transition band retains some residual absorbance after irradiation at 365 nm, as seen in Figure 7b, which suggests that *trans*-to-*cis* isomerisation is hindered in bulk, at least to some extent. This behaviour could not be explained, at least solely, by the greater concentration of azobenzene aggregates in the films, but also by the presence of interactions between the azobenzenes and other functional groups in the copolymers (AMPS and MMA) [52,63,64,65,66], which are both consistent with our model proposed in Figure 6.

### 2.5. Conductivity Response

**Figure 8:**
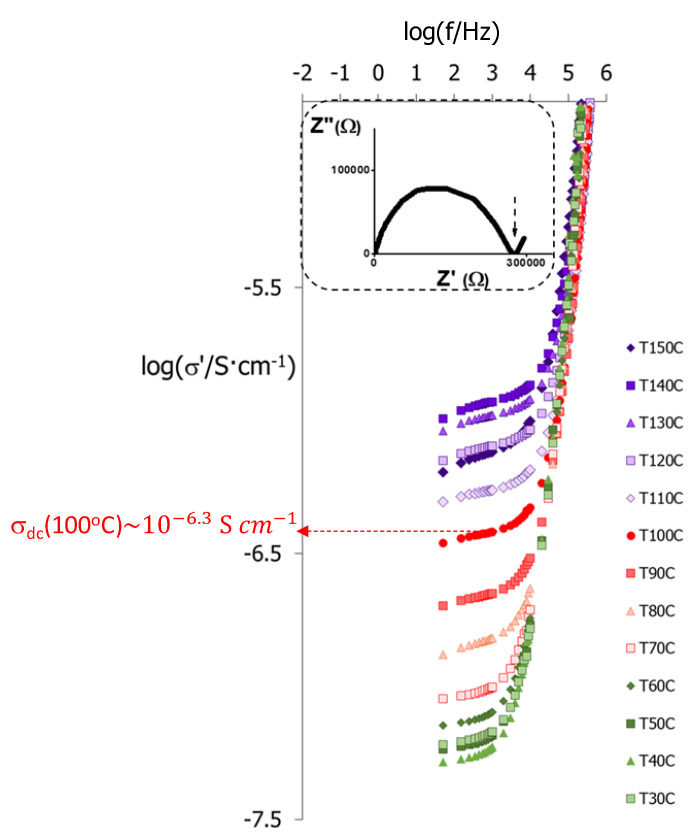
Double logarithmic plots of the real component, σ′, of the complex conductivity of PA-b-PS-b-PM, as a function of the frequency, measured in isothermal steps (°C) on cooling from the isotropic melt, and estimation of DC conductivity, σ_dc_ at T = 100 °C. Dotted arrow in the inset shows the spike in the impedance Nyquist plot, which is indicative of DC conductivity.

**Figure 9:**
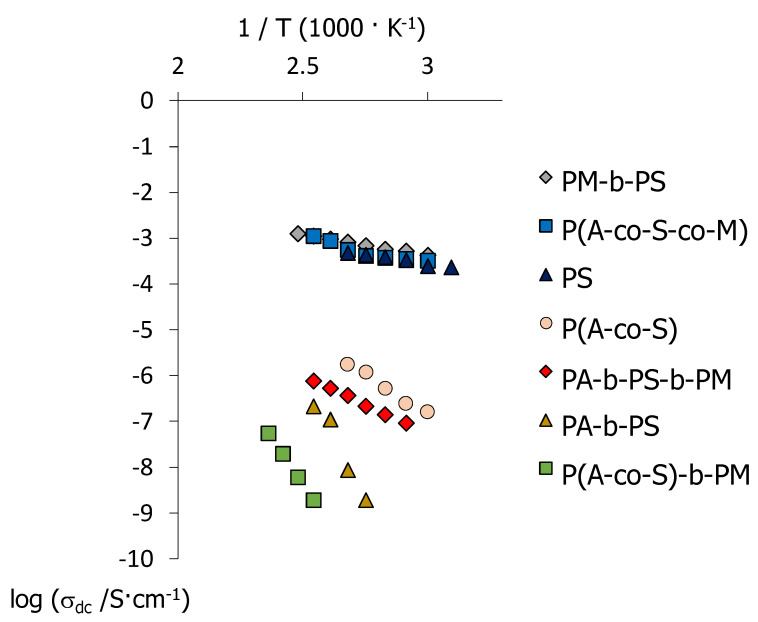
Arrhenius plots (in log_10_ scale) of the DC conductivity, σ_dc_, calculated for polymers containing AMPS polar groups.

We now investigate by impedance spectroscopy the effect of structure and composition on the conductivity of the copolymers, and in Figure 8 we illustrate their response with the isothermal σ′ curves obtained as a function of frequency, *f*, for the case of the triblock copolymer PA-b-PS-b-PM. The values of direct current (DC) conductivity, σ_dc_, can be obtained at each temperature from the plateaus in the log(σ′) *vs* log(*f*) curves in Figure 8, at sufficiently low frequencies, and correspond to the intersect with the real impedance axis, Z′, in Nyquist plots of the complex impedance $Z^{*}=Z^{\prime }+i\;Z^{\prime \prime }$ (see Figure 8 inset). The σ_dc_ values of all polymers in the series were estimated following a similar procedure, and the linear regions are summarised as a function of the temperature in Arrhenius plots in Figure 9. Table 4 depicts the activation energies, E_a_, calculated according to,

(1) $$\sigma_{dc}=\sigma_{0}\;\exp\left(\frac{E_{a}}{R}\;\frac{1}{T}\right)$$

where *R* is the gas constant, 8.31 J·mol^−1^·K^−1^; *T* is the absolute temperature; and σ_0_ is a pre-exponential term.

As expected, only polymers containing AMPS groups display well-defined plateaus in the double logarithmic σ′ vs *f* plots, and exhibit significant conductivity values, σ_dc_, since the sulfonic groups act as polar sites capable to transfer ionic groups from both electrodes, see Figure S7 [67]. The highest conductivities are observed for P(A-co-S-co-M) and PM-b-PS, which correspond to copolymers with the highest concentrations in AMPS groups, see Table 1, and fall in the 10^−3^ S cm^−1^ range, similarly to the PAMPS homopolymer, PS. We note, however, that none of these copolymers, PS, P(A-co-S-co-M) or PM-b-PS, are liquid crystalline.

PA-b-PS-b-PM and P(A-co-S), on the other hand, show appreciable conductivity values, in the 10^−6^ S cm^−1^ range, whilst keeping liquid crystalline behaviour. Even though these conductivity values are still below those displayed by benchmark polymer electrolytes, such as Nafion (~0.1 S cm^−1^) [25], we note that in the present work the conductivity was measured in anhydrous conditions, and our results are in good agreement with other liquid crystalline electrolytes [68,69,70,71,72,73]. Lastly, PA-b-PS and P(A-co-S)-b-PM display slightly lower conductivities, around ~10^−6.5^ S cm^−1^ and ~10^−8^ S cm^−1^, respectively.

In general terms, the conductivity has a positive correlation with the concentration of AMPS units in the copolymers, which bear the sulfonic groups involved in charge transport. The introduction of such polar groups, however, can have a disruptive effect on the anisotropic interactions between mesogenic units, and may ultimately inhibit liquid crystallinity [38,39,40,59]. Recently, we introduced methyl(methacrylate) groups to reduce potential interactions between AMPS and MeOAzB units, and we demonstrated that terpolymers containing near equimolar compositions, P(MeOAzB_0.29_-*co*-AMPS_0.36_-*co*-MMA_0.35_), can simultaneously yield significant conductivities (10^−4^ to 10^−7^ S·cm^−1^ range) and preserve liquid crystalline order [37]. We note that the statistical terpolymer prepared in the present work, P(A-co-S-co-M), presents high conductivities, but liquid crystallinity is inhibited due to the dilution of mesogenic units (18%, mol%).

**Figure 10:**
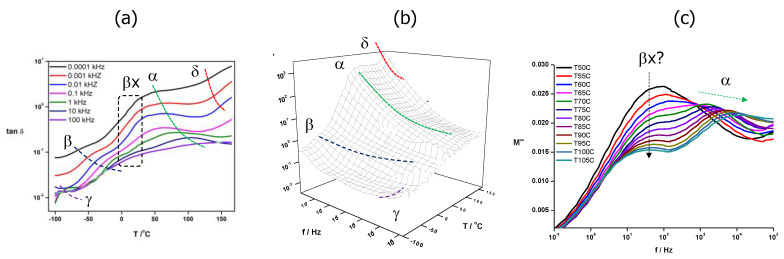
Dielectric response of PA-b-PS-b-PM, highlighting the δ-, α-, β-and γ- relaxations: (**a**) temperature dependence of tan δ = ε″/ε′ at selected frequencies; (**b**) 3D plot of the temperature and frequency dependence of the loss factor ε″; (**c**) imaginary component of the electric modulus, M″, as a function of the frequency, obtained at different temperatures. The appearance of a sub-glass βx process is also highlighted in the plots.

If we now return our attention to the triblock copolymer, PA-b-PS-b-PM, its conductivity values are considerably lower than those measured in this work for P(A-co-S-co-M), and slightly lower than those of P(MeOAzB_0.29_-*co*-AMPS_0.36_-*co*-MMA_0.35_) reported in [37], but PA-b-PS-b-PM has considerably lower concentration of sulfonic groups (20% AMPS, see Table 1). This result could be explained by the micro-segregation between mesogenic, polar and methyl(methacrylate) domains illustrated in Figure 6 for PA-b-PS-b-PM, and we have further studied the effect of the block structure on the conductivity by looking at the dielectric response of this triblock copolymer in Figure 10. More specifically, Figure 10a displays tan δ = ε″/ε′, as a function of the temperature and at selected frequencies, and Figure 10b summarises the dielectric response of this material in the full range of experimental temperatures and frequencies, via the dielectric loss factor, ε″.

Several relaxations of PA-b-PS-b-PM are visible in Figure 10, typical of comb-shape liquid crystalline polymers [74]. At low temperatures (−50 °C/0 °C), we discriminate a process that we attribute to the so-called β-relaxation, assigned to motions of the azobenzene groups in the MeOAzB chains [75,76]. At even lower temperatures and high frequencies, a secondary process seems to arise, which could be due to the γ-relaxation related to motions of the methylene spacer in MeOAzB, even though a broader experimental window would be necessary to confirm this assignation. These low-temperature relaxations are locally activated, and the exhibition of the β process is consistent with the preservation of the azobenzene response to light and electrical stimuli demonstrated above.

At higher temperatures, motions involving larger molecular segments are activated, and the α-relaxation is observed as a prominent process in the 50 °C/100 °C range in Figure 10, and related to the onset of main-chain segmental motions occurring in the vicinity of the glass transition, see Figure 3. At slightly lower temperatures, the α-relaxation seems to be accompanied and merged with another process, βx, which is particularly visible in the imaginary part of the electric modulus, M″, see Figure 10c. We hypothesise that this process could be assigned to the β1-relaxation, related to flip-flop motions of carbonyl groups near the polymer backbone of poly(methacrylate)s [77,78]. The very low temperature dependence of βx observed in Figure 10, however, cannot be explained by this assignation, and a more detailed dielectric study is necessary to determine its molecular origin.

At the high-temperature end in Figure 10b, the δ-relaxation is visible, involving the rotation of the MeOAzB side chains along the polymeric axis [75]. The onset of this relaxation has been attributed to the occurrence of long-range conductivity in comb-shape liquid crystalline polymers [37,74], and probably ion hopping between different smectic layers [79], facilitated by the large increase in free volume occurring near the liquid crystal to isotropic transition, T_LCI_. This seems to be the case for PA-b-PS and P(A-co-S)-b-PM, with σ_dc_ activation energies similar to those reported for δ-relaxations above 100 kJ mol^−1^, see Table 4 [75].

Conductivity in PA-b-PS-b-PM, on the other hand, occurs at lower temperatures and with lower activation energies (~48 kJ mol^−1^), suggesting that σ_dc_ may be activated by local motions. [74,80,81] Indeed, the activation energy of conductivity seems to be too low to be directly associated to the segmental motions of the α-relaxation, and, alternatively, it could have a similar molecular origin as the non-assigned βx-process, and being located at some specific sites near the polymer main chains. It is possible that the triblock structure may help disentangle local motions of the monomers, even though we note that it was difficult to discriminate by DSC between the glass transitions of each block (probably due to the small size of the domains discussed above). Local motions of sulfonic groups squeezed between MeOAzB domains in Figure 6 may then promote ionic conductivity in PA-b-PS-b-PM, ultimately achieving conductivity in the liquid crystal range (<120 °C), without the need for large free volumes involved in the δ-relaxation.

## 3. Materials and Techniques

AMPS and MMA were commercially available from Sigma-Aldrich (Saint Louis, MO, USA). MMA was purified by washing with sodium hydroxide and water, followed by drying with anhydrous magnesium sulfate; AMPS was used without further purification, and MeOAzB was prepared according to [82,83,84]. Different block and statistical copolymers were prepared by reversible addition-fragmentation chain polymerisation, RAFT [44], through the cascade of reactions illustrated in Figure 1. 4-cyano-4-[(dodecylsulfanylthiocarbonyl)sulfanyl]pentanoic acid (Aldrich, 97%, Saint Louis, MO, USA) was used as the chain transfer agent (CTA) and azobisisobutyronitrile (AIBN) as the initiator. Among the various living polymerisation methods, RAFT was selected due to the wide range of monomer selection and reaction conditions that it can tolerate. Further details of the materials and the synthetic procedure are included as Electronic Supplementary Information, ESI (Figure S1).

The chemical structures of the polymers and their intermediates were assessed by nuclear magnetic resonance, ^1^H-NMR. Number average molecular weights and weight average molecular weights (Mn and Mw, respectively), polydispersity (Mw/Mn) and degree of polymerisation (DP) of the polymers were obtained by gel permeation/size exclusion chromatography (GPC/SEC). The thermal stability of the materials was assessed by thermogravimetric analysis (TGA). The phase behaviour of the polymers was determined by differential scanning calorimetry (DSC) and phase identification was confirmed by polarised optical microscopy (POM). Phase structure was evaluated by X-ray diffraction (XRD) and small angle X-ray scattering SAXS, and the morphology of selected block copolymers was further assessed by transmission electron microscopy (TEM). Molecular lengths were estimated using ACD/ChemSketch software (Advanced Chemistry Development, Inc., ON, Canada).

The light response of the polymers was studied by UV-vis spectrophotometry. The ultraviolet-visible (UV-vis) absorbance spectra were recorded at room temperature, on films cast on quartz substrates, and on 3.73 × 10^−5^ M THF solutions, using a Perkin Elmer Lambda 750UV-VIS-NIR spectrometer (PerkinElmer Life and Analytical Sciences, Shelton, CT, USA) in the 200 to 800 nm wavelength range. The samples were measured before (ground state) and immediately after (excited state) being exposed for 10 min with UV light (wavelength = 365 nm, intensity = 50 µW·cm^−2^). Samples were then kept in the dark and were measured at various intervals until the spectra recovered its original shape (relaxation).

The ionic conductivity and the complex dielectric permittivity of the polymers were determined by impedance spectroscopy, in a broad range of temperatures and frequencies (from the isotropic to the glass phases).

Further information and details on the procedures and equipment used for the materials characterisation are included as supplementary information.

## 4. Conclusions

By using reversible addition-fragmentation chain transfer polymerization (RAFT), we have synthesised a series of side-chain copolymers including mesogenic azobenzenes (MeOAzB), sulfonic acid groups (AMPS) and non-mesogenic methyl(methacrylate) (MMA) groups, following statistical and block configurations, and with good control over composition, molecular weights, and polydispersities. The simultaneous introduction of the sulfonic and azobenzene groups has yielded liquid crystalline polymers containing polar sites and light-responsiveness.

Copolymers have ionic conductivities in the 10^−6^ S·cm^−1^ range, achieved with moderate concentrations of ionic groups (20%) and with liquid crystalline behaviour. The activation of ionic conductivity at low temperatures, and under anhydrous conditions, suggests that ionic mobility could be activated by local molecular motions near the polymeric backbone in AMPS-rich domains, potentially following ion hopping mechanisms.

We believe that the formation of different blocks with MeOAzB, AMPS and MMA units, distributed in narrow nano-segregated domains, opens the possibility to align the conductivity pathways by the application of anisotropic fields (mechanical shearing, light, electrical or magnetic-fields) by modifying contiguous liquid crystalline regions. The mechanisms to enhance local conductivity through specific blocks, and the formation of interconnected and aligned smectic domains, are the focus of ongoing investigations to optimise and integrate these materials as new liquid crystalline electrolytes in energy conversion and storage devices, but also as new components of sensors and actuators.

## Figures and Tables

**Table 1:** Polymer notation (P#) and full name, where the sub-indexes indicate the number of repeating units (for each block or macro-CTA) or molar fractions (within statistical segments); also included are number average molecular weight (Mw) and weight average molecular weight (Mn), polydispersities (Mw/Mn), yields and molar compositions of the three monomers. In bold, copolymers containing light-responsive (MeOAzB) and polar (AMPS) groups.

P#	Polymer Full Name	Mn g·mol^−1^	Mw g·mol^−1^	Mw/Mn	Yield (%)	MeOAzB/AMPS/MMA (% molar)
PA	PMeOAzB_25_^†^	11223	12920	1.15	68	100/0/0
PS	PAMPS_192_^†^	39863	73407	1.84	72	0/100/0
PM	PMMA_136_^†^	13613	15034	1.10	70	0/0/100
PA-b-PS	**PMeOAzB_25_-*b*-PAMPS_12_**	13705	24176	1.76	58	67.6/32.4/0
PA-b-PS-b-PM	**PMeOAzB_25_-*b*-PAMPS_12_-*b*-PMMA_23_**	16054	33817	2.10	52	41.7/20.0/38.3
P(A-co-S)	**P(MeOAzB_0.53_-*co*-AMPS_0.47_)_57_** ^‡^	19192	60525	1.83	71	53.0/47.0/0
P(A-co-S)-b-PM	**P(MeOAzB_0.53_-*co*-AMPS_0.47_)_57_-*b*-PMMA_119_** ^‡^	22901	57429	2.51	75	17.2/15.2/67.6
P(A-co-S-co-M)	**P(MeOAzB_0.18_-*co*-AMPS_0.47_-*co*-MMA_0.35_)** ^‡^	31088	89357	2.87	73	18.0/47.0/35.0
PA-b-PM	PMeOAzB_25_-*b*-PMMA_22_	13412	16060	1.22	55	53.2/0/46.8
PM-b-PS	PMMA_136_-*b*-PAMPS_471_	111356	124294	1.12	62	0/77.6/22.4

^†^ Macro CTAs. ^‡^ molar percentages were determined from ^1^H-NMR.

**Table 2:** Thermal parameters obtained from the DSC thermograms: glass transition, T_g_; liquid crystal to isotropic transition temperature, T_LCI_; enthalpy, ∆H_LCI_; and reduced entropy, ∆S_LCI_/R. All values obtained on second heating scans.

Polymer	T_g_ °C	T_LCI_ °C	∆H_LCI_ J·g^−1^	∆H_LCI_ kJ·mol^−1^	∆S_LCI_/R
PA	67.7	130.1	6.30	2.85	0.85
PS	138.0	-	-	-	-
PM	99.3	-	-	-	-
PA-b-PS	77.8	138.9	3.80	1.41	0.41
PA-b-PS-b-PM	77.6	138.9	2.31	0.73	0.21
P(A-co-S)	77.3	135.5	3.96	1.27	0.37
P(A-co-S)-b-PM	119.0	-	-	-	-
P(A-co-S-co-M)	59.0	-	-	-	-
PA-b-PM	69.6	131.7	2.40	0.91	0.27
PM-b-PS	110.0	-	-	-	-

**Table 3:** Summary of the main reflections obtained from the XRD and SAXS diffractograms, measured at room temperature (T = 25 °C). Colours indicate the tentative origin of the reflection.

P#	*d_i_* Å				
PA	17.58/15.81 ^†^	11.73			5.29
PS			10.85	7.00	*
PM				6.56	
PA-b-PS	15.35 ^†^		10.88	7.69	4.94 *
PA-b-PS-b-PM	15.25 ^†^		10.15	6.79	4.57 ^†^
P(A-co-S)	17.57 ^†^	11.51		8.64	5.42 ^†^
P(A-co-S)-b-PM				6.93 ^†^	
P(A-co-S-co-M)				7.30 ^†^	4.87 ^†^
PA-b-PM	17.48 ^†^	11.90 ^†^			5.37 ^†^
PM-b-PS				6.86 ^†^	

*—Growing peak at wider angles (2θ > 20°). ^†^—Measured by XRD.

**Table 4:** Activation energies, E_a_, of the direct current conductivity values, σ_dc_, obtained from the Arrhenius plots in Figure 9, and the corresponding temperature linear regions.

P#	E_a_ kJ mol^−1^	∆T Range °C
PS	16.1	50/100
PA-b-PS	198.2	90/120
PA-b-PS-b-PM	47.9	70/120
P(A-co-S)	65.7	60/100
P(A-co-S)-b-PM	156.2	120/150
P(A-co-S-co-M)	22.6	60/120
PM-b-PS	17.4	60/130

## Supplementary Materials

Supplementary Materials are available online.
