# Supplementary material for: Liquid Crystalline Copolymers Containing Sulfonic and Light-Responsive Groups: From Molecular Design to Conductivity
Source: Molecules. 2020 Jun 2;25(11):2579. doi: 10.3390/molecules25112579 (PMC7321325; doi:10.3390/molecules25112579)
Supplement: Supplementary file 1 [file molecules-25-02579-s001.pdf]

Electronic Supplementary Information

Liquid crystalline block copolymers containing sulfonic and light-responsive groups: from molecular design to conductivity.

Sakinah Mohd Alauddin [1,2], Nurul Fadhilah Kamalul Aripin [1,2], Thamil Selvi Velayutham[2,3] and Alfonso Martinez-Felipe [4,*]

[1] Faculty of Chemical Engineering, Universiti Teknologi MARA, 40450 Shah Alam, Selangor Darul Ehsan, Malaysia. sakinah3676@uitm.edu.my; fadhilah9413@uitm.edu.my.

[2]Fundamental and Frontier Sciences in Nanostructure Self-Assembly Center, Department of Chemistry, Faculty of Science, University of Malaya, 50603 Kuala Lumpur, Malaysia

[3]Low Dimensional Material Research Center, Department of Physics, Faculty of Science, University of Malaya, 50603 Kuala Lumpur, Malaysia. t_selvi@um.edu.my

[4] Chemical and Materials Engineering Research Group, School of Engineering, University of Aberdeen, King's College, Old Aberdeen AB24 3UE, UK. a.martinez-felipe@abdn.ac.uk.

* Correspondence: a.martinez-felipe@abdn.ac.uk; Tel.: +44(0) 1224 273 074.

Contents

1. Synthesis of materials……………………………………………………………………………………………p 3

Figure S1. Synthetic route used to yield PS, $PAMPS_{192}$; PM, $PMMA_{136}$; PM-b-PS, $PMMA_{136}$-*b*-$PAMPS_{471}$ and PA-b-PM, PMeOAzB $_{25}$-*b*-$PMMA_{22}$;

2. Techniques and methods……………………………………………………………………………………p 6

3. Results and discussion…………………………………………………………………………………… p 8

Figure S2. ^{1}H-NMR of P(A-co-S) ($CDCl_3$). Example of calculation of composition in statistical copolymers, using proton signals from the AMPS (a) and MeOAzB (b) units.

Figure S3. ^{1}H-NMR of polymers containing MeOAzB and AMPS groups: (a) PA, $PMeOAzB_{25}$; (b) PS, $PAMPS_{192}$; (c) PM, $PMMA_{136}$; (d) PA-b-PS, $PMeOAzB_{25}$-b-$PAMPS_{12}$; (e) PA-b-PM, PMeOAzB $_{25}$-*b*-$PMMA_{22}$; (f) PM-b-PS, $PMMA_{136}$-*b*-$PAMPS_{471}$; (g) PA-b-PS-b-PM, $PMeOAzB_{25}$-b-$PAMPS_{12}$-b-$PMMA_{23}$; (h) P(A-co-S), P($MeOAzB_{0.53}$-co-$AMPS_{0.47}$)$_{57}$; (i) P(A-co-S)-b-PM, P($MeOAzB_{0.29}$-co-$AMPS_{0.71}$)$_{57}$-b-$PMMA_{119}$; and (j) P(A-co-S-co-M), P($MeOAzB_{0.18}$-co-$AMPS_{0.47}$-co-$MMA_{0.35}$)

Figure S4. PA-b-PM polarised optical micrographs, POM, showing birefringence in the liquid crystal phase ($\mathrm{T} = 110$°C) and in the glass phase ($\mathrm{T} = 55$°C), obtained on cooling from the isotropic phase.

Figure S5. XRD of polymers containing MeOAzB and AMPS groups: : (a) PA, $PMeOAzB_{25}$; (b) PA-b-PS, $PMeOAzB_{25}$-b-$PAMPS_{12}$; (c) PA-b-PS-b-PM, $PMeOAzB_{25}$-b-$PAMPS_{12}$-b-$PMMA_{23}$; (d) P(A-co-S), P($MeOAzB_{0.53}$-co-$AMPS_{0.47}$)$_{57}$; (e) PA-b-PM, PMeOAzB $_{25}$-*b*-$PMMA_{22}$; (f) PS, $PAMPS_{192}$; (g) PM, $PMMA_{136}$; (h) P(A-co-S)-b-PM, P($MeOAzB_{0.29}$-co-$AMPS_{0.71}$)$_{57}$-b-$PMMA_{119}$; (i) P(A-co-S-co-M), P($MeOAzB_{0.18}$-co-$AMPS_{0.47}$-co-$MMA_{0.35}$); (j) PM-b-PS, $PMMA_{136}$-*b*-$PAMPS_{471}$

Figure S6. TEM of polymers PMeOAzB $_{25}$-*b*-$PMMA_{22}$, PA-b-PM, with different scales.

Figure S7. Double logarithmic isothermal plots of the real component, σ', of the complex conductivity, as a function of the frequency (*f*) obtained on cooling from the isotropic phase (T=150°C): (a) PS, $PAMPS_{192}$; (b) PM-b-PS, $PMMA_{136}$-*b*-$PAMPS_{471}$; (c) P(A-co-S-co-M), P(MeOAzB0.18-co-$AMPS_{0.47}$-co-$MMA_{0.35}$); and (d) P(A-co-S), P($MeOAzB_{0.53}$-co-$AMPS_{0.47}$)$_{57}$.

1. **Synthesis of materials**

Notation according to **Fig. 1** and **Table 1** (main manuscript).

PA, poly(10-(4-Methoxy-4′-oxy-azobenzene) decyl methacrylate) macro-CTA, $P(MeOAzB)_{25}$, was prepared by dissolving 4-cyano-4-[(dodecylsulfanylthiocarbonyl)sulfanyl]pentanoic acid, CTA, (3.4mg, 8.35 x10-3 mmol), MeOAzB (0.5g, 1.1 mmol) and AIBN (0.27mg, 1.67 $x10^{-3}$) mmol in 10 ml of anhydrous dimethylformamide in a clean dry Schlenk flask. The flask was then degassed with three freeze-pump-thaw cycles and flushed with nitrogen for 15 minutes. The reaction was conducted at 80°C for 24 hours, quenched with liquid nitrogen, diluted with dichloromethane and precipitated into cold diethyl ether. The polymer was further purified by dissolving in dichloromethane and precipitated in cold diethyl ether. ^{1}H NMR ($CDCl_3$, δ): 7.0, 7.9 (m, aromatic, 8 H), 4.1-3.9 (m, CH2OOC, OCH2, 4H), 3.8 (s, ArOCH3, 3H), 1.0-2.0 (m), 0.7-1.1 (CH3C(CO.O), 3 H).

PS, poly(2-acrylamido-2-methyl-1-propanesulfonic acid) macro-CTA, $PAMPS_{192}$, was prepared in an analogous manner as P1, where CTA (6.4mg, 0.016 mmol), 2-acrylamido-2-methyl-1-propanesulfonic acid (AMPS) (1g, 4.8 mmol), AIBN (0.53mg, 3.2 x10-3) mmol and 10 ml of dry dimethylformamide was placed in a dried schlenk flask. The flask was then subjected to three cycles of freeze-pump-thaw followed by flushing with nitrogen for 15 minutes. The reaction was conducted at 80°C for 24 hours, quenched with liquid nitrogen, diluted with dichloromethane and precipitated into cold diethyl ether. ^{1}H NMR (D_2O, δ): 1.5 (s, CH3, 3H), 2.1 (s, main chain CH2), 3.4 (s, CH2SO3H, 2H)

PM, polymethyl methacrylate macro-CTA, $PMMA_{136}$, was also prepared similar to P1 and P2, CTA (20mg, 0.05mmol), methyl methacrylate (1g, 10 mmol), AIBN (1.64mg, 0.01mmol) and 10ml of dry toluene was inserted into a dried schlenk flask and subjected to three cycles of freeze pump thaw and subsequent flushing with nitrogen. The reaction was heated to 80oC for 12 hours, quenched with liquid nitrogen, diluted with tetrahydrofuran and precipitated into cold methanol. ^{1}H NMR ($CDCl_3$, δ): 3.6 (s, OCH3), 1–2 (m, main chain CH2), 0.8-1.0 (CH3C(CO.O), 3 H).

PA-b-PS, poly(10-(4-Methoxy-4′-oxy-azobenzene) decyl methacrylate) -block- poly(2-acrylamido-2-methyl-1-propanesulfonic acid), $PMeOAzB_{25}$ -b- $PAMPS_{12}$, was synthesized by placing 100mg of P1, 250mg (1.2 mmol) of AMPS and 0.13 mg of AIBN in a clean Schlenk tube. 10 ml of dry DMF was then injected into the tube, subjected to three cycles of freeze, pump and thaw and finally flushed with nitrogen for 30min. The reaction was carried out at 80oC for 24 hour. The reaction was then quenched with liquid nitrogen and precipitated in diethyl ether to retrieve the polymer. The polymer was then further purified by dissolving in dichloromethane and precipitation in diethyl ether and dried in a vacuum oven overnight**.** ^{1}H NMR ($CDCl_3$, δ): 8.5 (s, NH,1H) 6.9, 7.9 (m, aromatic, 8 H), 3.9 (s, CH2-OOC, OCH2, 4H), 3.8 (s, ArOCH3, 3H), 2.8 (s, CH2-SO3, 2H) 1.0-2.0 (m), 0.7-1.1 (m, CH3C(CO.O), H main chain AMPS).

PA-b-PS-b-PM, poly(10-(4-Methoxy-4′-oxy-azobenzene) decyl methacrylate) -block- poly(2-acrylamido-2-methyl-1-propanesulfonic acid) -block- polymethyl methacrylate, $PMeOAzB_{25}$-b-$PAMPS_{12}$-b-$PMMA_{23}$. Was prepared by placing 40 mg of purified P4 di-block copolymer, 100 ml (0.2 mmol) of MMA and 0.1 mg of AIBN in a clean Schlenk tube. 10 ml of dry DMF was then injected into the tube, subjected to three cycles of freeze, pump and thaw and finally flushed with nitrogen for 30min. The reaction was carried out at 80°C for 24 hour. The reaction was then terminated in liquid nitrogen and diethyl ether was added to precipitate the polymer. The polymer was then purified by dissolving in dichloromethane and precipitation in diethyl ether and dried in a vacuum oven overnight. ^1H NMR ($CDCl_3$, δ): 8.5 (s, NH, 1H, AMPS) 7.0, 7.9 (m, aromatic, 8 H, MeOAzB), 4.1 – 3.9 (m, CH2-OOC, OCH2, 4H, MeOAzB), 3.8 (s, ArOCH3, 3H, MeOAzB), 3.6 (s, COO-CH3, 3H, MMA), 2.8 (s, CH2 – SO3, 2H, AMPS) 1.0 – 2.0 (m), 0.7 – 1.1 (m, CH3C(CO.O), H main chain)

P(A-co-S), poly(10-(4-Methoxy-4′-oxy-azobenzene) decyl methacrylate) -co- poly(2-acrylamido-2-methyl-1-propanesulfonic acid), $P(MeOAzB_{0.53}$-co-$AMPS_{0.47})_{57}$ was prepared by placing 14.4 mg (0.036 mmol) of CTA, 600mg (1.3 mmol) of 10-MeOAzB, 530 mg (2.6 mmol) of AMPS and 1.74 mg of AIBN in a clean Schlenk tube. 10 ml of dry DMF was then inserted into the tube and subjected to three cycles of freeze, pump and thaw with a final step of flushing with nitrogen. The reaction was carried out at 90°C for 24 hour. The reaction was then terminated using liquid nitrogen and subsequently precipitated in diethyl ether to retrieve the polymer. The polymer was then purified with dichloromethane and diethyl ether filtered and dried in a vacuum oven overnight. ^1H NMR ($CDCl_3$, δ): 8.5 (s, NH,1H) 6.9, 7.9 (m, aromatic, 8 H), 3.9 (s, CH2-OOC, OCH2, 4H), 3.8 (s, ArOCH3, 3H), 2.8 (s, CH2-SO3, 2H) 1.0-2.0 (m), 0.7-1.1 (m, CH3C(CO.O), H main chain AMPS).

P(A-co-S)-b-PM, poly[(10-(4-Methoxy-4′-oxy-azobenzene) decyl methacrylate) -co- poly(2-acrylamido-2-methyl-1-propanesulfonic acid)] -block- polymethyl methacrylate, $P(MeOAzB_{0.53}$-co-$AMPS_{0.47})_{57}$-b-$PMMA_{119}$ was synthesized by placing 50 mg of purified P8, 0.5 ml of MMA, 0.2 mg of AIBN and 10 ml of dry DMF in a clean dry Schlenk tube. The reaction tube was subjected to three cycles of freeze, pump and thaw with a final step of flushing with nitrogen. The reaction was carried out at 90°C for 24 hour. The reaction was placed in liquid nitrogen to terminate the reaction and the polymer was recovered with diethyl ether. The polymer was purified with dichloromethane and diethyl ether filtered and dried in a vacuum oven overnight. ^1H NMR ($CDCl_3$, δ): 8.5 (s, NH, 1H, AMPS) 7.0, 7.9 (m, aromatic, 8 H, MeOAzB), 4.1 – 3.9 (m, CH2-OOC, OCH2, 4H, MeOAzB), 3.8 (s, ArOCH3, 3H, MeOAzB), 3.6 (s, COO-CH3, 3H, MMA), 2.8 (s, CH2 – SO3, 2H, AMPS) 1.0 – 2.0 (m), 0.7 – 1.1 (m, CH3C(CO.O), H main chain)

P(A-co-S-co-M), poly(10-(4-Methoxy-4′-oxy-azobenzene) decyl methacrylate) -co- poly(2-acrylamido-2-methyl-1-propanesulfonic acid) -co- polymethyl methacrylate), $P(MeOAzB_{0.18}$-co-$AMPS_{0.47}$-co- $MMA_{0.35})$ was prepared with 14 mg CTA, 0.5 ml MMA, 600 mg of MeOAzB, 500 mg AMPS, 1.74 mg of AIBN and 10 ml of dry DMF was inserted. The reaction tube was subjected to three cycles of freeze, pump and thaw and a final step of flushing with nitrogen. The reaction was carried out at 90°C for 24 hour. The reaction was quenched in liquid nitrogen and the polymer was recovered in diethyl ether. The polymer was purified with dichloromethane and diethyl ether filtered and dried in a vacuum oven overnight. ^1H NMR ($CDCl_3$, δ): 8.5 (s, NH, 1H, AMPS) 7.0, 7.9 (m, aromatic, 8 H, MeOAzB), 4.1 – 3.9 (m, CH2-OOC, OCH2, 4H, MeOAzB), 3.8 (s, ArOCH3, 3H, MeOAzB), 3.6 (s, COO-CH3, 3H, MMA), 2.8 (s, CH2 – SO3, 2H, AMPS) 1.0 – 2.0 (m), 0.7 – 1.1 (m, CH3C(CO.O), H main chain)

PA-b-PM, poly(10-(4-Methoxy-4′-oxy-azobenzene) decyl methacrylate) -block- polymethyl methacrylate, $PMeOAzB_{25}$-b-$PMMA_{22}$ was synthesized similar to P4 where 50 mg of P1, 120 mg (1.23 mmol) of MMA and 0.23 mg of AIBN was weighed and placed in a clean Schlenk tube. 10 ml of dry DMF was then injected into the tube, subjected to three cycles of freeze, pump and thaw and finally flushed with nitrogen for 30min. The reaction was carried out at 80oC for 24 hour. The reaction was then terminated by liquid nitrogen and the polymer precipitated in diethyl ether. The polymer was then purified by dissolving in dichloromethane and precipitation in diethyl ether and dried in a vacuum oven overnight. ^{1}H NMR ($CDCl_3$, δ): 7.0, 7.9 (m, aromatic, 8 H), 4.1-3.9 (m, CH2OOC, OCH2, 4H), 3.8 (s, ArOCH3, 3H), 3.6 (s, OCH3), 1.0-2.0 (m), 0.7-1.1 (CH3C(CO.O), 3 H).

PM-b-PS, polymethyl methacrylate -block- poly(2-acrylamido-2-methyl-1-propanesulfonic acid), $PMMA_{136}$-b-$PAMPS_{471}$, was prepared with by weighing 50mg of purified P3, 110 mg (0.54 mmol) of AMPS and 0.2 mg of AIBN and subsequently placed in a clean Schlenk tube. 10 ml of dry DMF was then injected into the tube, subjected to three cycles of freeze, pump and thaw and finally flushed with nitrogen for 30min. The reaction was carried out at 80°C for 24 hour. The reaction was then terminated by liquid nitrogen and diethyl ether was added to precipitate the polymer. The polymer was then purified by dissolving in dichloromethane and precipitation in diethyl ether and dried in a vacuum oven overnight. ^{1}H NMR ($CDCl_3$, δ): 8.5 (s, NH), 3.6 (s, OCH3), 2.8 (s, CH2-SO3, 2H) 1.0-2.0 (m), 0.7-1.1 (m, CH3C(CO.O), H main chain AMPS).

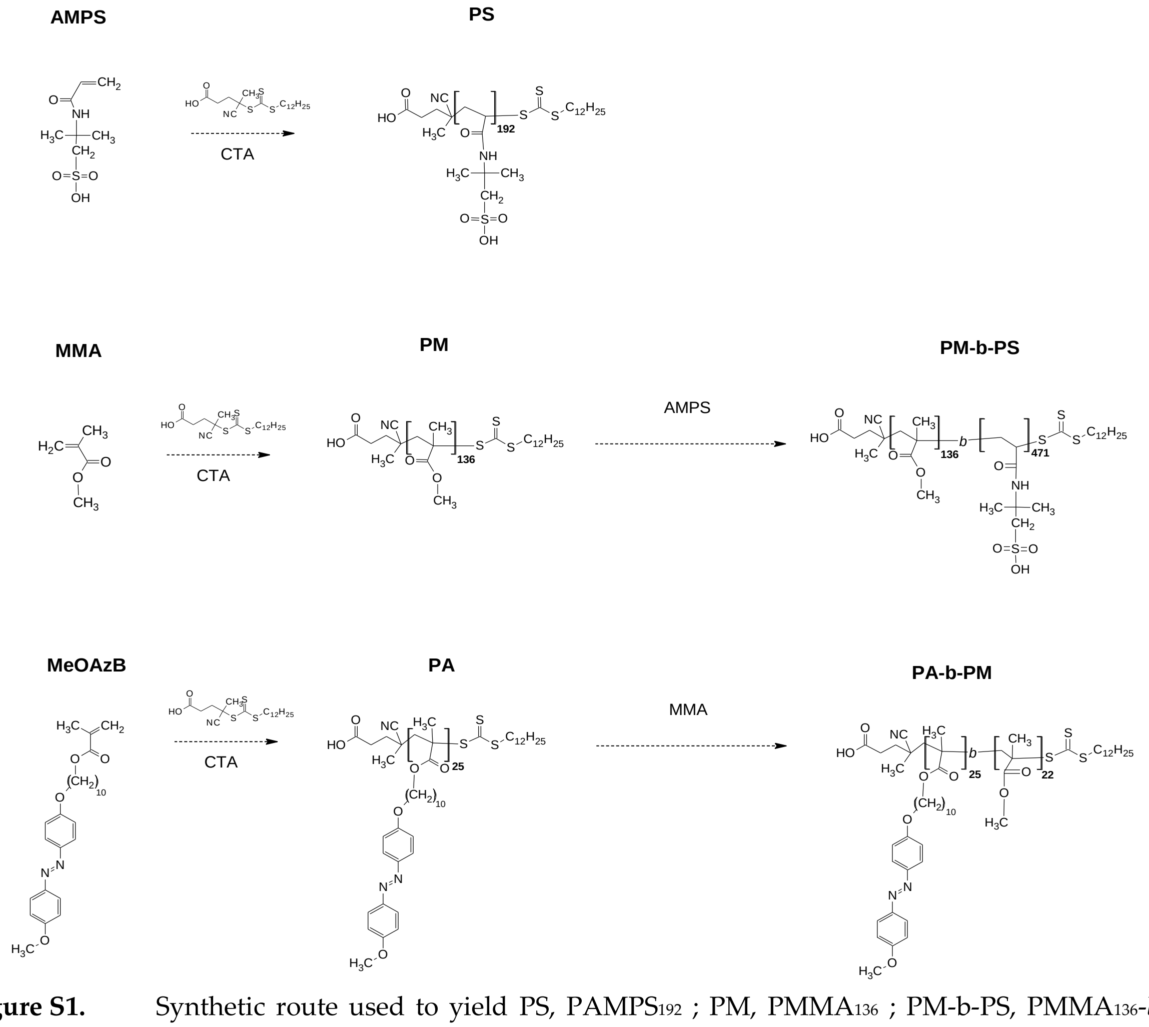

Figure S1. Synthetic route used to yield PS, $PAMPS_{192}$; PM, $PMMA_{136}$; PM-b-PS, $PMMA_{136}$-*b*-$PAMPS_{471}$ and PA-b-PM, $PMeOAzB_{25}$-*b*-$PMMA_{22}$;

2. Techniques and methods

The chemical structures of the polymers and their intermediates were assessed by nuclear magnetic resonance, ^{1}H-NMR, using a 400 MHz Bruker AVANCE III NMR spectrometer, and by infrared spectroscopy, IR, using a Perkin Elmer Spectrum One FT-IR spectrometer equipped with an Attenuated total reflectance (ATR). For the ^{1}H-NMR experiments, deuterated chloroform ($CDCl_3$) or deuterium oxide (D_2O) was used as the solvent. IR measurements were taken at room temperature, on KBr dispersions of the samples (~1%, in weight %), using pristine KBr discs as the background. The IR spectra were obtained in the 4000 – 400 cm^{-1} range, with a 4 cm^{-1} accuracy, as the average of 64 scans. The average molecular weights, Mw and Mn, polydispersity, Mw/ Mn, and degree of polymerisation, DP, of the polymers, were measured using a Water 1515 module with a Waters 2414 refractive index detector . Samples were eluted in tetrahydrofuran with a flow rate of 1 ml·min^{-1} at 25°C. Polystyrene standards were used for calibration.

The phase behaviour of the polymers was determined by differential scanning calorimetry, DSC, using a Mettler Toledo DSC1 module. Around 4 mg of sample were heated from 25°C to 200°C, held at 200°C for 3 minutes, cooled to 25°C, held for 3 minutes, and then reheated again to 200°C. All scans were conducted at a rate of ±10°C·min^{-1} under nitrogen atmosphere. Phase identification was confirmed by an Olympus BX51 polarised optical microscope, POM. The thermal stability of the materials was assessed by thermogravimetric analysis, TGA, using a Mettler Toledo TGA/DSC1 module from 25°C to 800°C, under 10 ml·min^{-1} flowrate of nitrogen.

X-ray diffraction, XRD, patterns were obtained using a Aeris,PANalytical X-ray diffractometer equipped with CuKα-radiation filtered by cross-coupled Göbel mirrors at 40 kV and 40 mA. Samples were heated to 150℃, cooled slowly to room temperature, and their diffraction pattern recorded. The small angle X-ray scattering, SWAXS, were conducted by placing the dried sample in a paste cell with colourless window. The SAXSpace (Anton Paar) instrument was equipped with an X-ray tube (DX-Cu 12x0.45, SERFERT) generating Cu-Kα with wavelength l = 1.542 Å at 40 kV and 50 mA. The Goebel-mirror focused and Kratky-slit collimated X-ray beam was line shaped with a 17 mm horizontal dimension at the sample, and scattered radiation from the samples, which is measured in transmission mode, was recorded with a one- dimensional MYTHEN-1k microstrip solid-state detector (Dectris Switzerland) within a 2θ angle of 0° to 20°. Silver behenate was used as the standard reference. The measurement was run at various temperature using a temperature controlled sample stage (TCStage 300). Molecular lengths were estimated using ACD/ChemSketch. The morphology of some block copolymers was further assessed by transmission electron microscopy, TEM, using TEM (Tecnai G2 20). The polymer samples were initially heated to isotropic, cooled to room temperature and embedded in an epoxy resin before sectioning using an Leica EM UC6 ultramicrotome. The sliced sample were placed on a carbon grid, stained with 2% uranyl acetate before viewing.

Dielectric measurements were carried out on metal-insulator-metal (MIM) structure devices. Firstly, the polymers were dissolved in dichloromethane (DCM), to yield 5 % weight solutions, and glass substrates were then pre-cleaned by sonicating for 10 min in soap water, acetone, isopropanol and distilled water, and dried with nitrogen gas. The clean glasses were then pre-coated with aluminium *via* thermal evaporation, and the DCM solution were spin coated on the substrate to yield homogeneous transparent thin films, which was dried in an oven at 80°C for 24 hours, in order to remove residual solvent. The thickness of the resulting films was in the range 1.5 ± 0.1 μm, measured by a KLA Tencor P-6 mechanical profilometer. Aluminium top electrodes were then deposited on the films to produce the MIM structure devices, which were subsequently used for dielectric analyses. Measurements were carried out in the frequency range of 0.01 Hz to 1 MHz, by using a combination of two apparatus: a home-made dielectric spectrometer (0.01–10^4 Hz), and an Agilent 4294A impedance analyser (10^2–10^6

Hz). The spectra were obtained in isothermal steps on heating from T=-100°C to 165°C, and the isothermal frequency sweeps were performed after the corresponding temperature was allowed to stabilise for 10 min.

Ionic conductivity was measured using a Hioki 3532-50 LCR Hi tester in the frequency range of 50 Hz to 5 MHz, between 303 and 423 K. A few mg of the polymers were melted on stainless steel electrodes (20 mm diameter, SUS316), allowed to cool slowly into their liquid crystal phases, covered with a second similar electrode, and then further cooled to room temperature. The electrodes were separated by a Teflon ring of internal diameter 12 mm. Samples were heated above their clearing point inside the cell, and then measured in isothermal steps on cooling to room temperature. The results were obtained in terms of the complex dielectric permittivity, $\varepsilon^* = \varepsilon' - i\varepsilon''$, which was transformed into the complex impedance, Z^*, and conductivity, σ^*, using:

$$Z^* = Z' + iZ'' = \frac{1}{i\omega C_0 \varepsilon^*}$$

and

$$\sigma^* = i\ \omega \varepsilon_0 \varepsilon^*$$

where i is the imaginary unit, ω is the angular frequency in rad · s^{-1}, C_0 is the cell capacitance and ε_0 is the permittivity in the vacuum, $8.854x10^{-12}F\ m^{-1}$.

3. Results and discussion

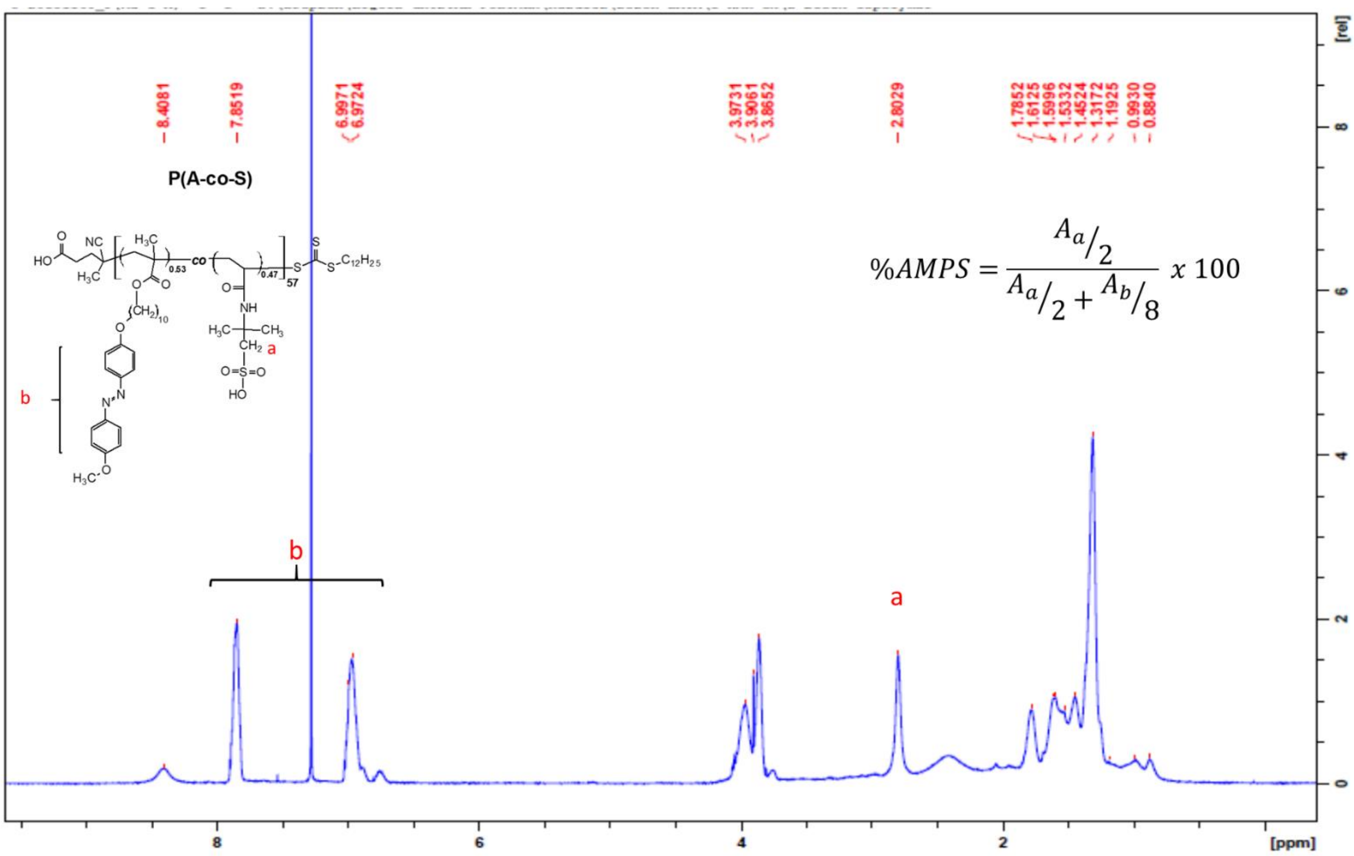

Figure S2. ^{1}H-NMR of P(MeOAzB$_{0.53}$-co-AMPS$_{0.47}$)$_{57}$, P(A-co-S) ($CDCl_3$). Example of calculation of composition in statistical copolymers, using proton signals from the AMPS (a) and MeOAzB (b) units.

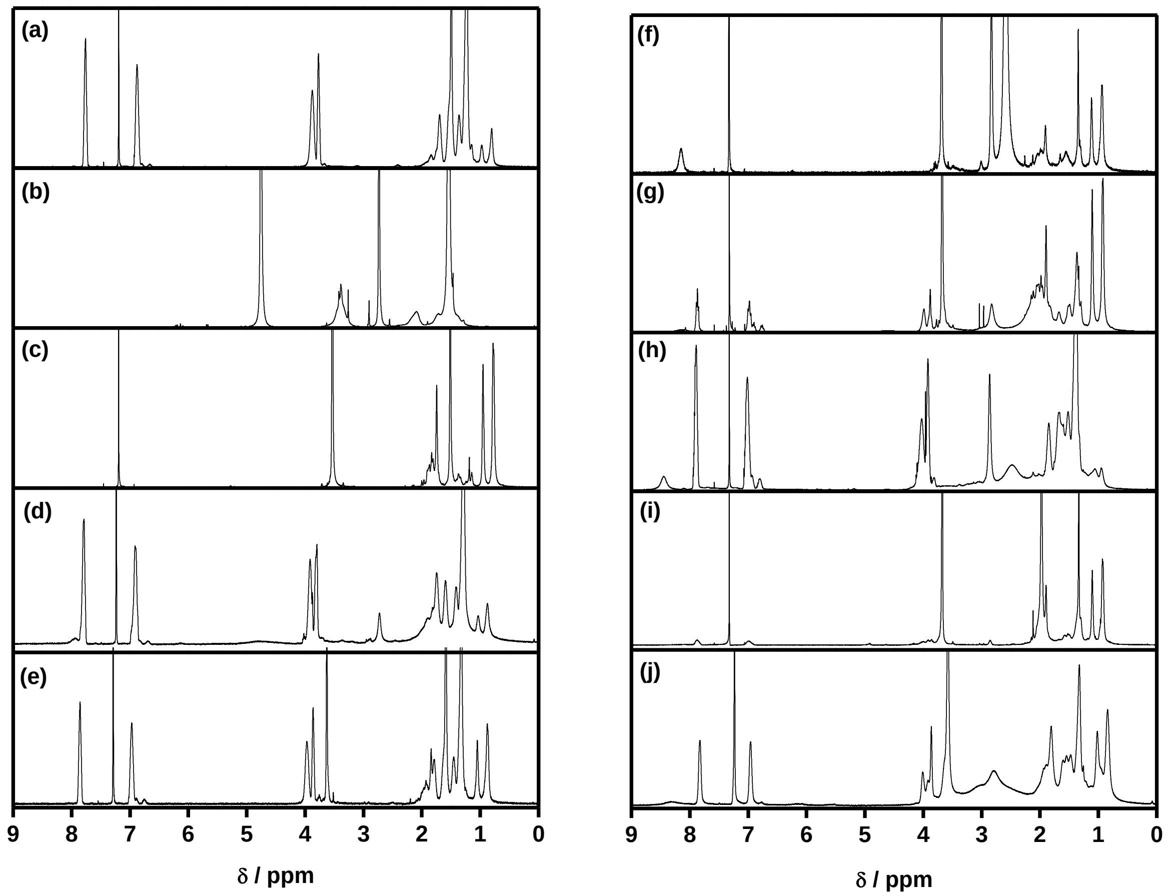

Figure S3. ^{1}H-NMR of polymers containing MeOAzB and AMPS groups: (a) PA, PMeOAzB$_{25}$; (b) PS, PAMPS$_{192}$; (c) PM, PMMA$_{136}$; (d) PA-b-PS, PMeOAzB$_{25}$-b-PAMPS$_{12}$; (e) PA-b-PM, PMeOAzB$_{25}$-*b*-PMMA$_{22}$; (f) PM-b-PS, PMMA$_{136}$-*b*-PAMPS$_{471}$; (g) PA-b-PS-b-PM, PMeOAzB$_{25}$-b-PAMPS$_{12}$-b-PMMA$_{23}$; (h) P(A-co-S), P(MeOAzB$_{0.53}$-co-AMPS$_{0.47}$)$_{57}$; (i) P(A-co-S)-b-PM, P(MeOAzB$_{0.29}$-co-AMPS$_{0.71}$)$_{57}$-b-PMMA$_{119}$; and (j) P(A-co-S-co-M), P(MeOAzB$_{0.18}$-co-AMPS$_{0.47}$-co-MMA$_{0.35}$)

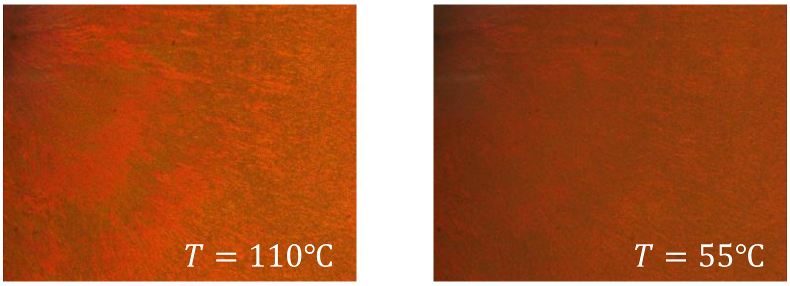

Figure S4. PA-b-PM polarised optical micrographs, POM, showing birefringence in the liquid crystal phase (T = 110℃) and in the glass phase (T = 55℃), obtained on cooling from the isotropic phase.

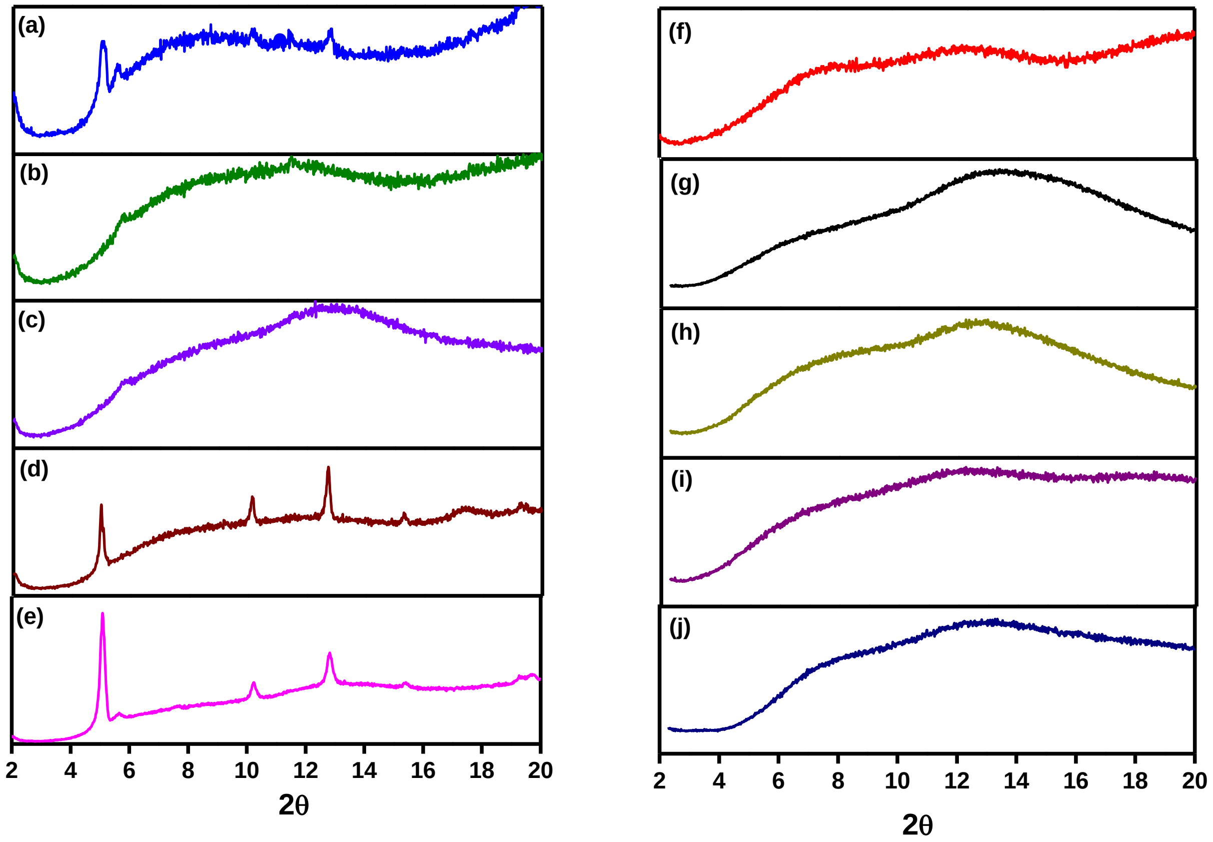

Figure S5. XRD of polymers containing MeOAzB and AMPS groups: : (a) PA, $PMeOAzB_{25}$; (b) PA-b-PS, $PMeOAzB_{25}$-b-$PAMPS_{12}$; (c) PA-b-PS-b-PM, $PMeOAzB_{25}$-b-$PAMPS_{12}$-b-$PMMA_{23}$; (d) P(A-co-S), $P(MeOAzB_{0.53}$-co-$AMPS_{0.47})_{57}$; (e) PA-b-PM, $PMeOAzB_{25}$-*b*-$PMMA_{22}$; (f) PS, $PAMPS_{192}$; (g) PM, $PMMA_{136}$; (h) P(A-co-S)-b-PM, $P(MeOAzB_{0.29}$-co-$AMPS_{0.71})_{57}$-b-$PMMA_{119}$; (i) P(A-co-S-co-M), $P(MeOAzB_{0.18}$-co-$AMPS_{0.47}$-co-$MMA_{0.35})$; (j) PM-b-PS, $PMMA_{136}$-*b*-$PAMPS_{471}$

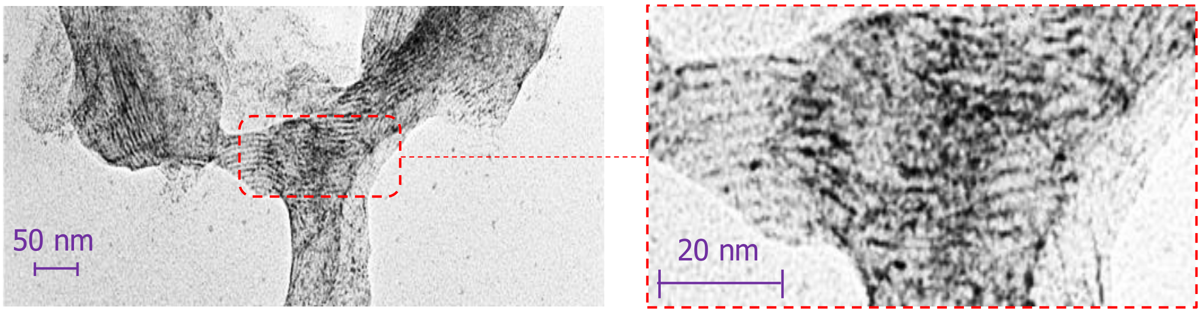

Figure S6. TEM of polymers $PMeOAzB_{25}$-*b*-$PMMA_{22}$, PA-b-PM, with different scales.

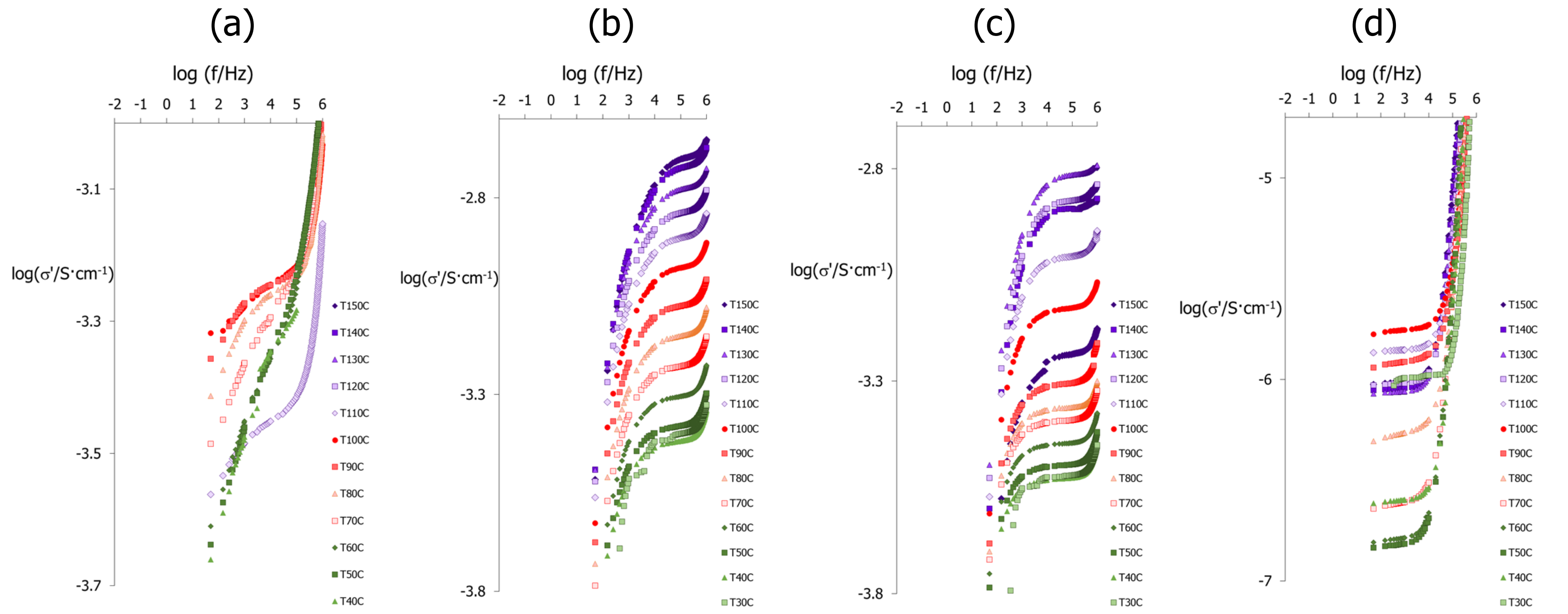

Figure S7. Double logarithmic isothermal plots of the real component, σ′, of the complex conductivity, as a function of the frequency (*f*) obtained on cooling from the isotropic phase (T=150°C): (a) PS, PAMPS$_{192}$; (b) PM-b-PS, PMMA$_{136}$-*b*-PAMPS$_{471}$; (c) P(A-co-S-co-M), P(MeOAzB0.18-co-AMPS$_{0.47}$-co-MMA$_{0.35}$); and (d) P(A-co-S), P(MeOAzB$_{0.53}$-co-AMPS$_{0.47}$)$_{57}$.
